# Urgent distal pancreatectomy for intraperitoneal hemorrhage due to the spontaneous rupture of a pancreatic metastatic tumor from synovial sarcoma: a case report

**DOI:** 10.1186/s12893-020-00832-6

**Published:** 2020-08-05

**Authors:** Takahiro Yokose, Minoru Kitago, Go Oshima, Kodai Abe, Yohei Masugi, Eisuke Miura, Masahiro Shinoda, Hiroshi Yagi, Yuta Abe, Shutaro Hori, Yohji Matsusaka, Yutaka Endo, Kenji Toyama, Shigeo Okuda, Yuko Kitagawa

**Affiliations:** 1 Department of Surgery, Keio University School of Medicine, Shinanomachi 35, Shinjuku-ku, Tokyo, 160-8582 Japan; 2 Department of Pathology, Keio University School of Medicine, Shinanomachi 35, Shinjuku-ku, Tokyo, 160-8582 Japan; 3 Department of Radiology, Keio University School of Medicine, Shinanomachi 35, Shinjuku-ku, Tokyo, 160-8582 Japan

**Keywords:** Synovial sarcoma, Metastatic pancreatic tumor, Spontaneous rupture, Distal pancreatectomy, Intraperitoneal hemorrhage, Case report

## Abstract

**Background:**

Synovial sarcoma is a soft tissue malignancy that frequently affects the extremities, adjacent to the large joints. Synovial sarcoma has a high rate of distant metastasis; however, pancreatic metastasis is extremely rare, and to our knowledge, there has been no report of bleeding due to spontaneous tumor rupture. This study reports the case of a patient with synovial sarcoma pancreatic metastasis causing tumor rupture and bleeding, which was successfully managed with emergent distal pancreatectomy.

**Case presentation:**

A 27-year-old woman underwent extensive resection of the primary tumor and partial lung resection after chemotherapy for left femoral synovial sarcoma and multiple lung metastases 4 years prior. During the follow-up, a 35-mm tumor was noted in the pancreatic tail on abdominal computed tomography (CT), and no other distant metastases were detected via positron emission tomography CT. Laparoscopic distal pancreatectomy was scheduled for pancreatic metastasis of synovial sarcoma. However, before the scheduled pancreatectomy could be conducted, the patient visited the emergency department because of abdominal pain that occurred after consuming a small amount of alcohol, and CT showed ascites with high CT values and leakage of contrast media. She was diagnosed with intra-abdominal hemorrhage due to a ruptured metastatic pancreatic tumor, and an emergency operation was performed. In total, 1500 mL of blood was evacuated from the abdomen, and the bleeding pancreatic tail tumor was resected. Histopathological findings revealed synovial sarcoma metastasis and a ruptured tumor capsule, and tumor cells were observed in the hematoma. After discharge on postoperative day 18, the patient was carefully monitored and confirmed to be in relapse-free survival, without chemotherapy, at 6 months post-surgery.

**Conclusions:**

While the rate of tumor growth varies depending on the grade of the tumor, the possibility of rupture should be considered even in metastatic pancreatic tumors. In the case of pancreatic tumor rupture with stable circulation, radiological evaluation for oncology is necessary, and primary resection may be compatible with resectable cases.

## Background

The spontaneous rupture of a pancreatic tumor is an uncommon occurrence, although ruptured cystic pancreatic tumors, solid pseudopapillary neoplasms (SPN), and intraductal papillary mucinous neoplasms have been reported [1–6]. Metastatic tumors to the pancreas account for approximately 2% of all pancreatic tumors [7], and spontaneous rupture of metastatic pancreatic tumors is extremely rare [8]. Synovial sarcoma is a high-grade neoplasm, characterized by significant local invasiveness and metastatic tendency, and accounts for 10–20% of soft tissue sarcomas in the adolescent and young adult population [9]. Incidence peaks of synovial sarcomas are observed in the first 30 years of life; these tumors affect the extremities, adjacent to the large joints [10]. The main treatment is extensive surgical excision combined with adjuvant or neoadjuvant radiation therapy, which provides a favorable prognosis for localized disease. However, the disease tends to recur both early and late, and the 10-year disease-free survival rate remains approximately 50% [10]. Synovial sarcomas have a high rate of distant metastases, most of which are observed in the lungs, followed by the bones, lymph nodes, and skin. Pancreatic metastasis is extremely rare, and to our knowledge, there have been no reports of bleeding caused by tumor rupture. In the case of pancreatic tumor rupture into the abdominal cavity and bleeding, urgent management is required, and surgery is a viable option. Here, we provide the first report of a patient with synovial sarcoma pancreatic metastasis causing tumor rupture and bleeding, which was successfully treated with distal pancreatectomy.

## Case presentation

The patient was a 27-year-old woman who first visited our hospital 4 years prior because of pain in the left femur. She was diagnosed with a left femoral synovial sarcoma with simultaneous multiple lung metastases. After chemotherapy, the primary lesion and lung metastasis decreased, and she underwent extensive resection of the primary lesion and lung metastases. During follow-up, abdominal computed tomography (CT) revealed a mass in the pancreatic tail. Before that time, only a chest CT was performed, and the abdomen was not evaluated. No notable hematology abnormalities were observed, and tumor markers (carcinoembryonic antigen: 0.7 ng/mL and carbohydrate antigen: 19–9: 7 U/mL) were within the normal ranges.

**Fig. 1:**
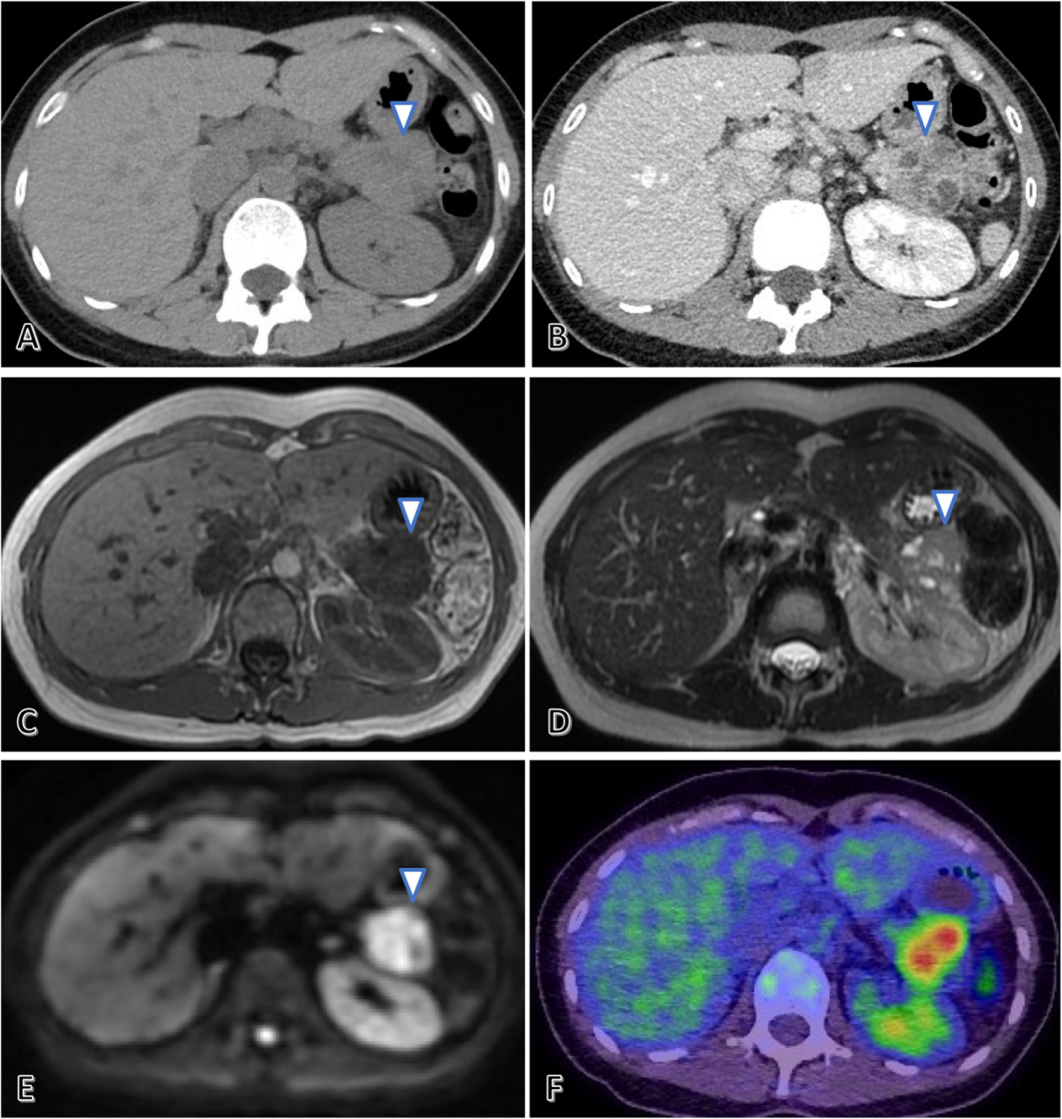
Radiological findings of the pancreatic tail tumor. Non-contrast abdominal computed tomography (CT) reveals a large, 35-mm tumor in the pancreatic tail without calcification (arrow) (**a**). Contrast-enhanced CT shows that the tumor had heterogeneous contrast enhancement without bleeding (arrow) (**b**). Abdominal magnetic resonance imaging reveals that the pancreatic tail tumor (arrow) presented as a low signal area on the T1-weighted image (**c**), a slightly hyperintense tumor containing multiple cystic components on the T2-weighted image (**d**), and a high signal area on the diffusion-weighted image (**e**). Positron emission tomography CT indicates abnormal fluorine-18 fluorodeoxyglucose accumulation only in the pancreatic tail tumor (**f**)

Contrast-enhanced thoracoabdominal CT revealed a large, 35-mm tumor in the pancreatic tail (Fig. 1a, b) showing heterogeneous contrast enhancement without calcification or bleeding. Lymphadenopathy and distant metastases were not observed.

Abdominal magnetic resonance imaging (Fig. 1c–e) revealed that the tumor in the pancreatic tail was similar to that in the primary left femoral lesion, showing a low signal on the T1-weighted image, a slightly hyperintense tumor containing multiple cystic components on the T2-weighted image, and a high signal on the diffusion-weighted image. The inside of the pancreatic tail tumor was heterogeneous and partly showed a water signal that was indicative of necrosis. ^18^F-fluorodeoxyglucose positron emission tomography CT revealed abnormal fluorodeoxyglucose accumulation only in the tumor of the pancreatic tail, with no other metastatic findings (Fig. 1f).

The preoperative diagnosis was synovial sarcoma recurrence of pancreatic tail metastasis, and laparoscopic distal pancreatectomy was planned. However, 2 weeks after the outpatient consultation, she developed left upper quadrant abdominal pain at night after drinking a small amount of alcohol, and she visited our emergency department. Her vital signs were as follows: pulse, 66 beats/min; respiration, 18 breaths/min; blood pressure, 100/60 mmHg; and temperature, 36.8 °C. Physical examination revealed tenderness and rebound pain in the left upper quadrant of the abdomen. Hematology findings revealed leukocytosis (13,400 cells/mm^3^) and anemia (hemoglobin: 11.0 g/dL).

**Fig. 2:**
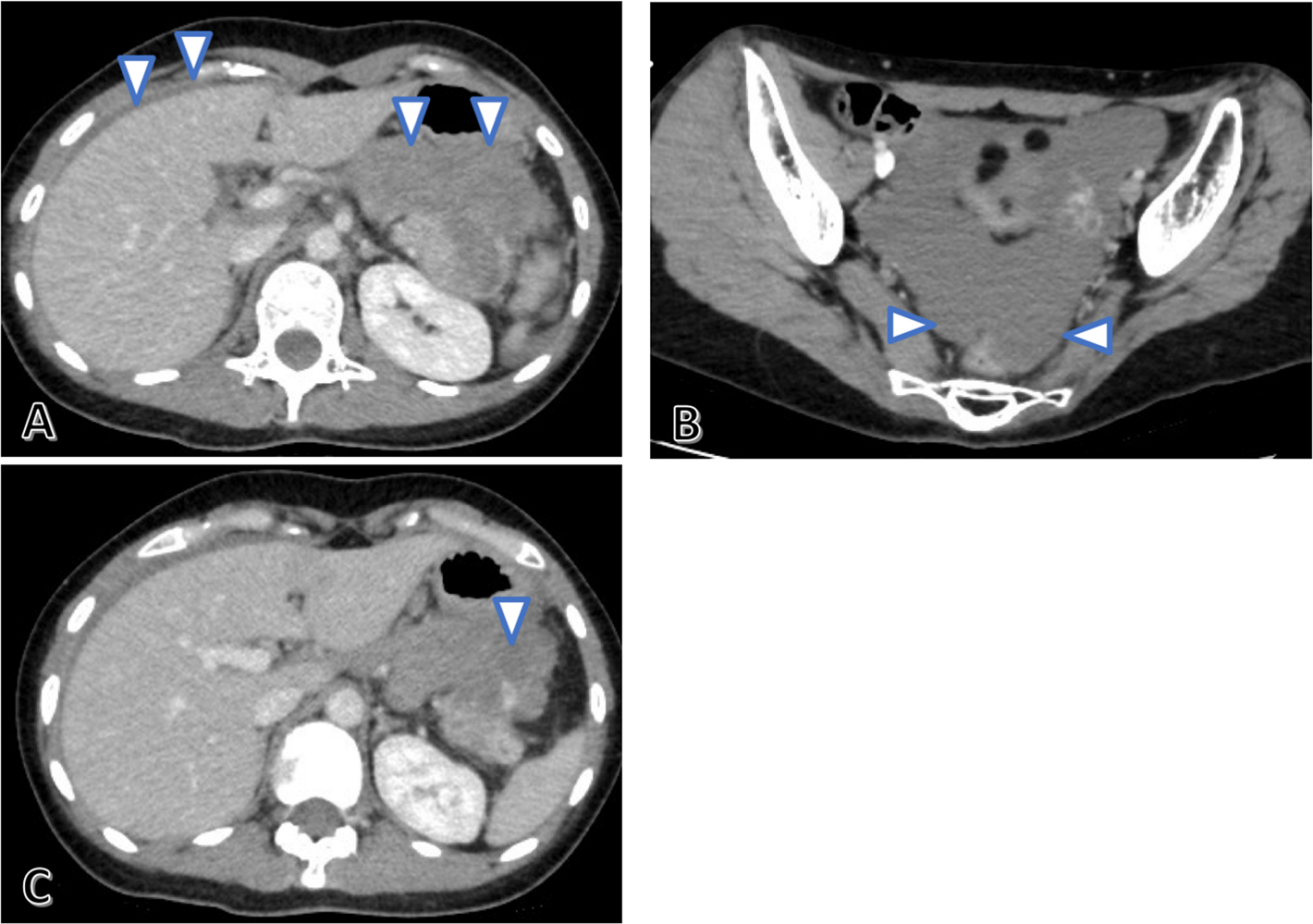
Contrast-enhanced abdominal computed tomography (CT) conducted in the emergency department. Ascites with high CT values (arrow) is observed on the liver surface (**a**) and in the pelvis (**b**). Leakage of contrast medium (arrow) is observed from the anterior of the pancreatic tail tumor to the omental bursa (**c**)

Contrast-enhanced abdominal CT performed in the emergency department revealed ascites with high CT values in the liver surface and pelvis (Fig. 2a, b), and leakage of contrast medium in the anterior of the pancreatic tail tumor to the omental bursa (Fig. 2c). The diagnosis was intra-abdominal hemorrhage due to a ruptured metastatic pancreatic tail tumor, and an urgent distal pancreatectomy was, therefore, performed.

**Fig. 3:**
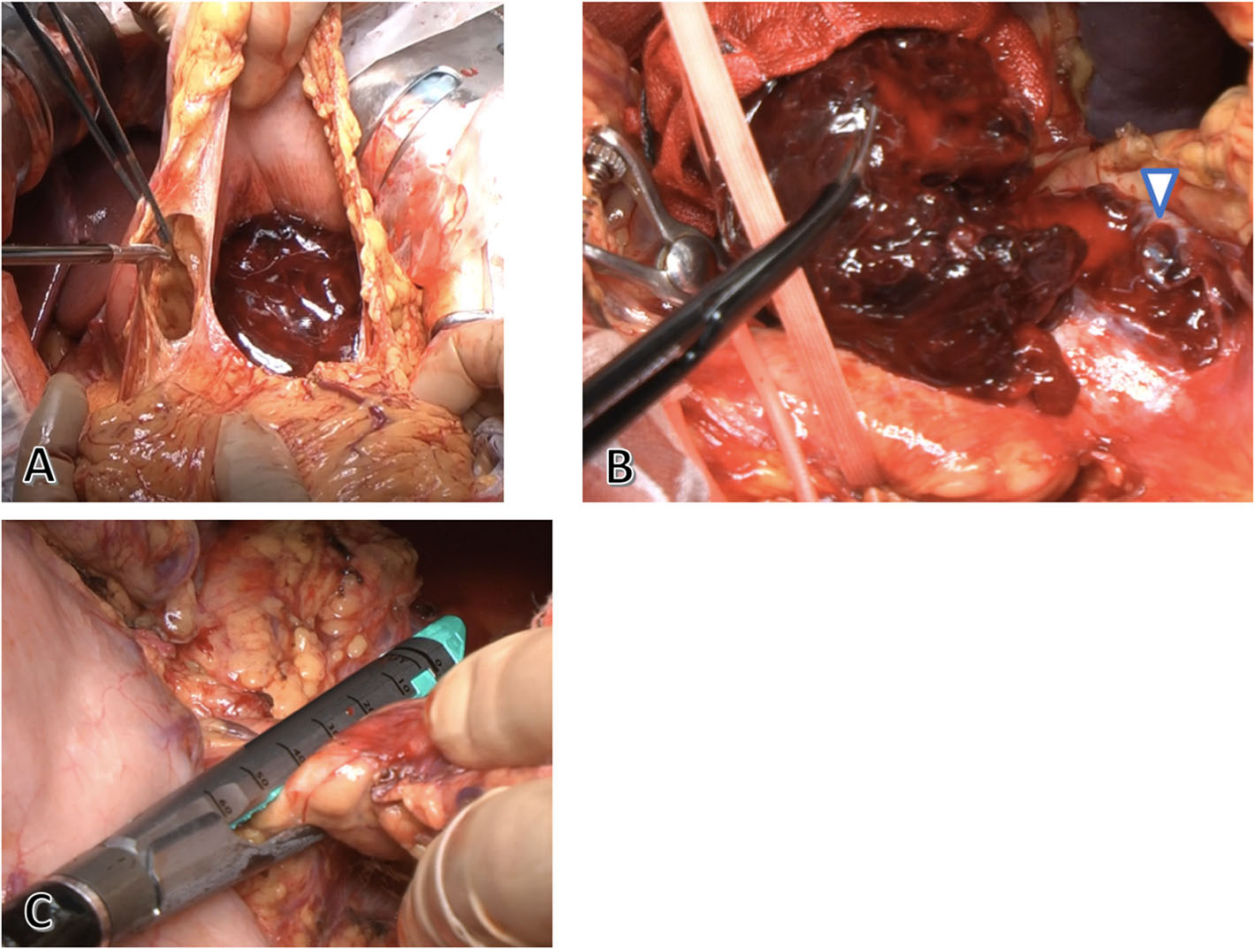
Urgent operation findings. A hematoma is observed during laparotomy and on the omentum bursa (**a**). Bleeding from the tumor capsule of the pancreatic tail is identified after opening the omentum (arrow) (**b**). The pancreas is dissected with linear stapling devices over 15 min (**c**)

During the emergency surgery, approximately 1500 mL of hematoma was observed during laparotomy, and bleeding from the tumor of the pancreatic tail was identified after opening the omentum (Fig. 3a, b). The splenic artery and vein were isolated and individually ligated to control the bleeding. The pancreas was dissected with linear stapling devices for over 15 min, and distal pancreatectomy was performed (Fig. 3c). The operation time was 123 min, including the hematoma suctioning, and the amount of bleeding was 1530 mL; the patient required four units of concentrated red blood cell transfusion.

Ascites cytology was performed during the emergency surgery and after surgery, and the results were negative.

According to the International Study Group of Pancreatic Fistula classification, a pancreatic fistula of the grade “biochemical leak” was observed, and she was discharged on postoperative day 18.

**Fig. 4:**
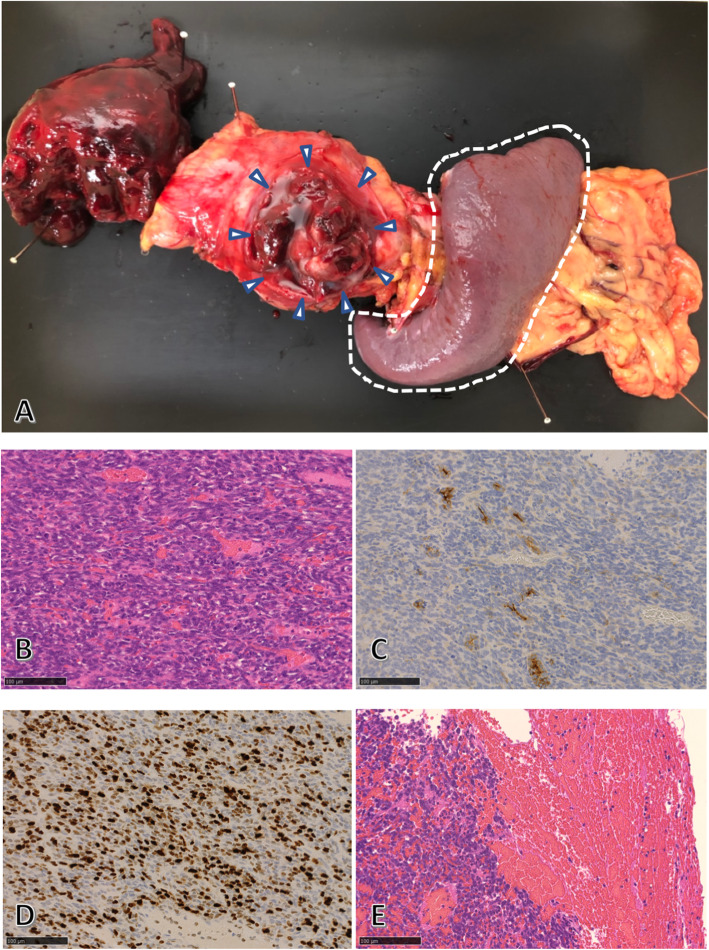
Pathological findings. Surgical specimens of the pancreas show the ruptured tumor capsule of the pancreatic tail tumor. A hematoma is visible on the left of the specimen. The white arrowheads indicate the tumor, and the spleen is outlined with the white dotted line (**a**). Histological findings of the specimen reveal monophasic spindle cells with a high nuclear/cytoplasmic ratio. The tumor contains stag horn-like vessels (**b**). Some of the tumor cells tested positive for epithelial membrane antigen (**c**). The tumor shows a high MIB-1 index (**d**). Tumor cells are observed within the intra-abdominal hematoma due to tumor rupture (**e**). Hematoxylin and eosin staining (**b** and **e**)

Analysis of the resected specimen showed that the capsule of the pancreas tail tumor was ruptured, and the tumor parenchyma was exposed (Fig. 4a). Upon histological examination, hematoxylin and eosin staining revealed the presence of monophasic spindle cells that grew solidly with stag horn-like vessels. Immunohistochemistry revealed that the tumor cells were positive for B-cell lymphoma 2 and CD99 but lacked expression of CD34, alpha-smooth muscle actin, desmin, and S100. Some of the tumor cells tested positive for epithelial membrane antigen. The MIB-1 proliferation index identified via MIB-1 staining was approximately 70%, indicating a high-grade sarcoma, and the tumor was compatible with synovial sarcoma metastasis to the pancreas (Fig. 4b–e). The resection margin of the pancreas was negative; however, tumor cells were exposed from the capsule owing to tumor rupture, and tumor cells were observed in the hematoma.

Regarding the follow-up after discharge, the patient has been carefully monitored without chemotherapy, because the target lesion has been previously resected, and confirmed to be in relapse-free survival for 6 months.

## Discussion and conclusions

Pancreatic tumor rupture includes rupture due to trauma and spontaneous tumor rupture. Spontaneous rupture of pancreatic tumors is an unusual course. There are some reports of spontaneous rupture of primary pancreatic tumors, such as cystic pancreatic tumors, SPN, and intraductal papillary mucinous neoplasms [1–6]. Some pregnancy-related cystic pancreatic tumors have the potential to grow and rupture during pregnancy [1, 11]. In patients with pregnancy-related cystic pancreatic tumors, ruptured tumors tended to be larger than non-ruptured tumors (13.5 ± 4.9 cm vs. 9.8 ± 1.1 cm) [11].

In the case of rupture of a malignant pancreatic tumor that was originally indicated for resection, curative resection is required even after non-operative management. Ruptured tumors that spread into the abdominal cavity, often reported as hepatocellular carcinoma rupture, have a poor prognosis [12]. In case of pancreatic tumor rupture, if the circulation is unstable, damage control surgery should be planned; however, in an event it is stable, radiological evaluation for oncology is necessary, and primary resection may be compatible for resectable cases. En bloc tumor resection without rupture or exposure of the tumor is important. Regarding rupture, mucinous cystic carcinoma and SPN reportedly have poor prognosis or increased recurrence [13, 14].

Tumors that metastasize to the pancreas include pancreatic cancer and other organ malignancies [15, 16]. Tumor metastasis from other organs to the pancreas is rare and occurs in approximately 2% of pancreatic tumors [7]. The primary site of the resected tumor is mostly renal cell carcinoma, followed by lung cancer, gastrointestinal cancer, breast cancer, melanoma, lymphoma, and soft tissue tumors, such as sarcoma [7, 17–19]. To the best of our knowledge, this is the first report to describe a case of spontaneous rupture of pancreatic metastasis from synovial sarcoma.

Complete resection with a margin is important for the treatment of synovial sarcoma [20]. Multidisciplinary treatment combined with radiotherapy and chemoradiotherapy is important in unresectable cases or those with positive margins [21]. Recently, the molecular mechanism underlying translocation-related sarcoma has been elucidated, and biological target treatments are being developed [22]. The prognosis of synovial sarcoma is moderately poor, and distant metastases occur in approximately 50% of patients [23]. Pancreatic metastasis of synovial sarcoma is extremely rare, and only four cases have been reported to date [24–27]. Furthermore, there are no reports of hemorrhage due to the rupture of pancreatic metastatic tumors in patients with synovial sarcoma. Table 1 summarizes the four cases of pancreatic metastasis that were previously reported along with this case. Two patients with resected sporadic pancreatic metastases experienced no recurrence for more than 30 months; however, the prognosis was unknown in two patients who did not undergo surgical resection because of extrapancreatic metastases. Complete resection can be expected to improve prognosis in cases with sporadic pancreatic metastasis of synovial sarcoma. As in this case, control of bleeding is necessary, and life-saving emergency surgery is appropriate for a ruptured tumor capsule and bleeding. In this case, tumor cells were observed in the hematoma at the time of urgent surgery; however, the use of adjuvant chemotherapy in such cases is controversial. The first- and second-line chemotherapies have been previously completed for the advanced metastatic synovial sarcoma. Radiological findings showed no target lesion after the pancreatic tumor was resected, causing difficulty in the evaluation of the effectiveness of chemotherapy. Due to these reasons, the patient was followed up frequently without chemotherapy. Although the tumor was resected with negative margins, strict follow-up is essential in cases with tumor rupture. This report was limited by a short observation period of only 6 months.

**Table 1:** Characteristics of reported cases and the present case of pancreatic metastasis from synovial sarcoma

Author	Year	Sex	Age, years	Duration from primary to pancreatic metastasis, years	Number of pancreatic metastases	Size, cm	Location	Extra pancreatic metastasis	Rupture	Treatment	Prognosis
Yamamoto et al. [24]	2001	Female	40	14	1	N/A	Head	No	No	PPPD	DFS > 6 years
Patel et al. [25]	2006	Female	44	10	1	8	Head	Yes	No	Biliary drainage	N/A
Krishna et al. [26]	2014	Male	38	1	8	0.3–1.9	Head–Tail	Yes	No	N/A	N/A
Makino et al. [27]	2016	Male	36	4	1	3.5	Body	No	No	Laparoscopic DP	DFS 30 months
Present case	2020	Female	27	4	1	3.5	Tail	No	Yes	Urgent DP	DFS 6 months

Abbreviations: *N/A* not available; *PPPD* pylorus preserved pancreaticoduodenectomy; *DP* distal pancreatectomy; *DFS* disease-free survival

In conclusion, while the rate of tumor growth varies depending on the grade of the tumor, the possibility of rupture should be considered even in metastatic pancreatic tumors. In the case of pancreatic tumor rupture with stable circulation, radiological evaluation for oncology is necessary, and primary resection may be compatible with resectable cases.

## Data Availability

Not applicable.

## Abbreviations

CT: Computed tomography
SPN: Solid pseudopapillary neoplasm
